# Supplementary figures and images for: Modulation of the gut microbiota by processed food and natural food: evidence from the Siniperca chuatsi microbiome
Source: PeerJ. 2024 Jun 14;12:e17520. doi: 10.7717/peerj.17520 (PMC11182020; doi:10.7717/peerj.17520)

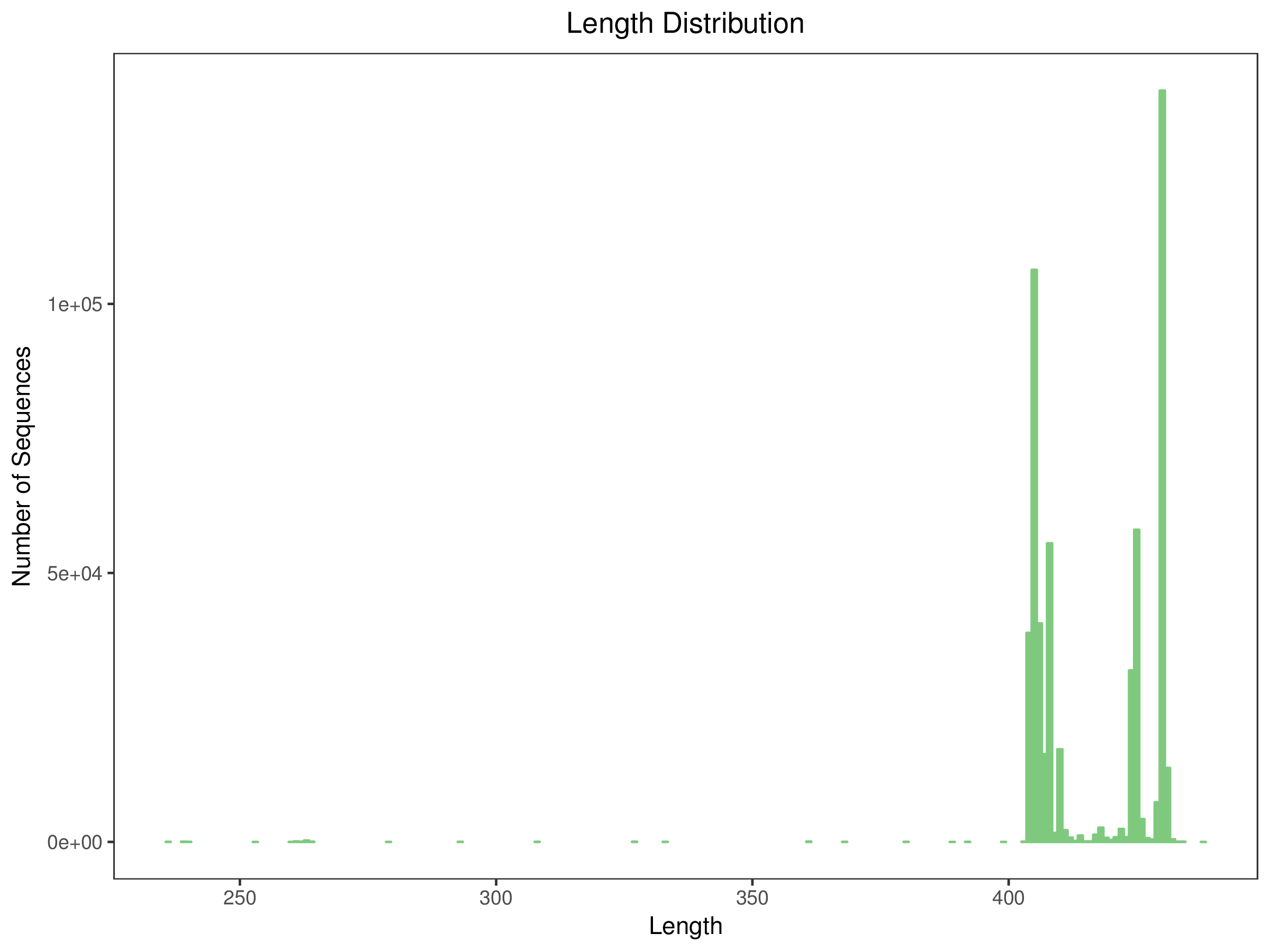

Supplement: Supplemental Information 1 [file peerj-12-17520-s001.png]

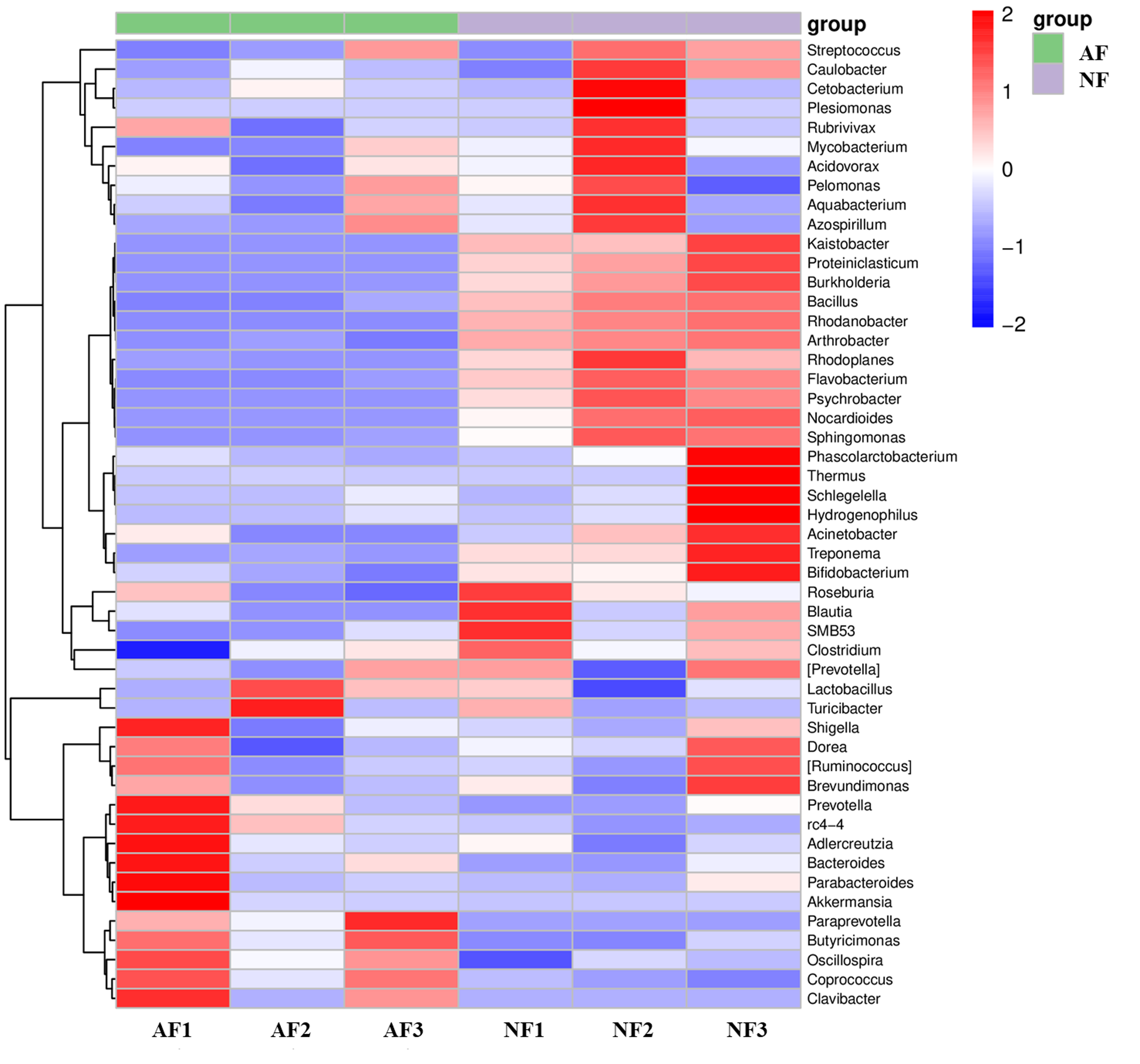

Supplement: Supplemental Information 2 [file peerj-12-17520-s002.png]
