# Supplementary material for: Modulation of the gut microbiota by processed food and natural food: evidence from the Siniperca chuatsi microbiome
Source: PeerJ. 2024 Jun 14;12:e17520. doi: 10.7717/peerj.17520 (PMC11182020; doi:10.7717/peerj.17520)
Supplement: Supplemental Information 3 [file peerj-12-17520-s003.docx]

**Supplementary Table S1** Chemical compositions of the FB and AF diets.

| **Chemical composition (%)** | | **NF** | **AF** |
| --- | --- | --- | --- |
| Crude protein | 14.14 | | 51.69 |
| Crude lipid | 1.50 | | 9.70 |
| Carbohydrate | 1.26 | | 13.81 |
| Moisture | 78.80 | | 6.10 |
| Ash | 4.30 | | 18.70 |
