# Supplementary material for: Modulation of the gut microbiota by processed food and natural food: evidence from the Siniperca chuatsi microbiome
Source: PeerJ. 2024 Jun 14;12:e17520. doi: 10.7717/peerj.17520 (PMC11182020; doi:10.7717/peerj.17520)
Supplement: Supplemental Information 4 [file peerj-12-17520-s004.docx]

**Supplementary Table S2 The16 S Sequencing data of gut microbiota in mandarin fish fed with the FB and AF diet**

| **SampleID** | **Input** | **Filtered** | **Denoised** | **Merged** | **Non-chimeric** | **Non-singleton** |
| --- | --- | --- | --- | --- | --- | --- |
| NF1 | 207043 | 194875 | 188075 | 171862 | 134731 | 133265 |
| NF2 | 124786 | 116184 | 111672 | 101051 | 90445 | 89419 |
| NF3 | 126116 | 117862 | 113648 | 103938 | 94128 | 93212 |
| AF1 | 121590 | 113960 | 111310 | 105404 | 88729 | 88069 |
| AF2 | 108012 | 101015 | 98439 | 92130 | 74042 | 73473 |
| AF3 | 93073 | 87264 | 84712 | 79602 | 66331 | 65764 |
